# Does chest compression during cardiopulmonary resuscitation provide sufficient cerebral oxygenation?

**DOI:** 10.3906/sag-1809-165

**Published:** 2019-02-11

**Authors:** Mehmet Akif YAZAR, Mehmet Barış AÇIKGÖZ, Adnan BAYRAM

**Affiliations:** 1 Konya Training and Research Hospital, Meram, Konya Turkey; 2 Nevşehir Public Hospital, Nevşehir Turkey; 3 Faculty of Medicine, Erciyes University, Kayseri Turkey

**Keywords:** Cardiopulmonary resuscitation, chest compression, neurological outcome

## Abstract

**Background/aim:**

Some of the patients suffering from cardiac arrest (CA) remain in a chronic unconscious state in intensive care units (ICUs). The primary aim of this study was to evaluate the efficacy of chest compression (CC) on cerebral oxygenation during cardiopulmonary resuscitation (CPR). As a secondary goal, we attempted to determine the effects of regional cerebral oxygen saturation (rSO2) values on consciousness and the survival rate using the Full Outline of Unresponsiveness (FOUR) scoring method.

**Materials and methods:**

This observational preliminary study was carried out with 20 patients with CA who were hospitalized in ICUs. The rSO2 values measured by near-infrared spectroscopy were recorded during CA. FOUR scoring was used to determine the neurological status, severity of disease, and degree of organ dysfunction in survivors.

**Results:**

Return of spontaneous circulation (ROSC) was gained in 8 (40%) of 20 patients. Maximum rSO2 values were higher in survivors than in nonsurvivors (P = 0.005). The mean FOUR score before CA was 11.50 ± 0.8 in survivors, whereas this value was 7.87 ± 0.7 for 1 week after ROSC (P < 0.0001). There was a significant positive correlation between the minimum and mean rSO2 values and the mean 1-week FOUR scores in survivors (r = 0.811, r = 0.771 and P = 0.015, P = 0.025, respectively).

**Conclusion:**

Our results suggest that the maximum rSO2 values affect ROSC while the minimum and mean rSO2 values affect the post-cardiac arrest neurological outcome.

## 1. Introduction

When brain perfusion is impaired, an irreversible process begins for neurons within about 5–8 min (1). Impairment of brain perfusion during cardiac arrest (CA) almost always causes reduction of cerebral oxygenation, resulting in neurological damage (2). If the first intervention cannot be fast, or if adequate chest compression (CC) cannot be provided during cardiopulmonary resuscitation (CPR), sufficient cerebral oxygenation cannot be obtained. This is closely related to chronic unconsciousness or minimal consciousness state in cases of return of spontaneous circulation (ROSC) (3). It is defined that the threshold for ischemia is ≤50% or 20% decrease from the baseline value (4). Cerebral oximetry achieved by near-infrared spectroscopy (NIRS) technology provides information about a level of brain oxygenation and cerebral perfusion (5). NIRS is also used as a noninvasive monitoring technique that demonstrates the quality of CPR (6). Studies have shown that high regional cerebral oxygen saturation (rSO2) values measured by NIRS during CA are consistent with the ROSC rate (7). 

Despite frequent updates of international CPR guidelines, trained healthcare providers still perform inadequate CC with low cerebral perfusion pressure (8). Some researchers showed that the quality of CC correlated with poor neurologic outcomes (9). For this reason, some patients remain in a chronic unconscious state in intensive care units (ICUs) and the discharge rates are low (10). Although survival rates are better after CPR in hospital CA cases, a comprehensive study suggested that only 17% of these patients were discharged alive (11).

The primary aim of this study was to evaluate the efficacy of CC on cerebral oxygenation during CPR. As a secondary goal, we attempted to determine the effects of rSO2 values on the survival rate and consciousness status of patients with ROSC using the Full Outline of Unresponsiveness (FOUR) scoring method. 

## 2. Materials and methods

### 2.1. Patient selection and approval

This observational preliminary study was approved by the Erciyes University Clinical Research Ethics Committee (ClinicalTrials.gov: NCT03062306) and carried out with 20 patients with CA who were hospitalized in the ICU of Nevşehir Public Hospital from May 2016 to August 2017. Informed consent was waived due to the emergency situation. All intubated patients with agonal status due to illness and ≥18 years of age who were followed in the ICU were included in the study.

### 2.2. NIRS application and rSO2 recording

Procedures related to the study did not disrupt routine CPR practice. Patients with CA in the ICU were monitored with an NIRS device (INVOS 5100c, Somanetics, Troy, MI, USA) during CPR. Bilateral frontal noninvasive sensors of the device were applied on the frontal area of the patient. The device continuously recorded the right and left rSO2 values, but we wrote down the values at 60-s intervals. The rSO2 recording was continued until the CPR was terminated in non-ROSC patients, while it was continued for 10 min after ROSC in the patients with ROSC. The rSO2 values used in the study were averages of bifrontal right and left rSO2 values.

### 2.3. Cardiopulmonary resuscitation

CPR was performed by doctors or nurses in the ICU. All patients with CA were treated with advanced life support (ALS) in accordance with the 2015 guidelines of the European Resuscitation Council. Since the patients were followed in the ICU, they were already monitored by three-lead electrocardiography (ECG), pulse oximeter, and noninvasive blood pressure devices. All our patients had a fast onset of CPR due to follow-up in the ICU and CPR was started as soon as cardiac arrest occurred because they were being consistently monitored in the ICU. We defined the patients with ROSC as survivors. 

### 2.4. FOUR scoring

FOUR scoring tests four neurological parameters. The number of components and the maximum score in each of the categories is four. FOUR collects all the requirements of a neurological consciousness examination and offers much more neurological detail (12). The FOUR scoring is shown in Table 1. We used FOUR scoring to determine the neurological status, the severity of disease, and the degree of organ dysfunction in patients with ROSC. FOUR scores were recorded for 1 week by a physician in the ICU once a day.

**Table 1:** The FOUR scale.

Eye response			
Eyelids open or opened, tracking or blinking to command	4	
Eyelids open but not tracking	3	
Eyelids closed but open to loud voice	2	
Eyelids closed but open to pain	1	
Eyelids remain closed with pain	0
Motor response		
Thumbs-up, fist, or peace sign	4
Localizing to pain	3
Flexion response to pain	2
Extension response to pain	1
No response to pain, or generalized myoclonus status	0
Brainstem reflexes		
Pupil and corneal reflexes present	4
One pupil wide and fixed	3
Pupil or corneal reflexes absent	2
Pupil and corneal reflexes absent	1
Absent pupil, corneal and cough reflex	0
Respiration			
Not intubated, regular breathing pattern	4	
Not intubated, Cheyne–Stokes breathing pattern	3	
Not intubated, irregular breathing	2	
Breathes above ventilator rate	1	
Breathes at ventilator rate or apnea	0

FOUR: Full Outline of Unresponsiveness.

### 2.5. Exclusion criteria

Patients were excluded from the study if they had cranial events such as cranial trauma, intracranial hemorrhage, or cerebral ischemic vascular; pulmonary diseases that may affect oxygenation such as pneumonia or chronic obstructive pulmonary disease (COPD); FOUR scores lower than 10 before CA; or age under 18 years.

### 2.6. Statistical analysis

Statistical analyses were performed using SPSS for Windows 16.0 (SPSS Inc., Chicago, IL, USA). Data with normal distribution were presented as mean ± standard deviation. The paired sample t-test was used to evaluate the significance of continuous variable parameters. Pearson’s correlation analysis was used for normal distribution to determine the correlation between numerical variables. Correlation coefficient (r) was evaluated as follows: 0.0001 to 0.249 is poor; 0.250 to 0.499 is moderate; 0.500 to 0.749 is strong; 0.750 to 1.000 is a very strong relationship. P < 0.05 was considered statistically significant.

## 3. Results

ROSC was gained in 8 (40%) of 20 patients who consisted of 8 (40%) males and 12 (60%) females. The mean age was 72.6 ± 4.2 years in survivors and 77.3 ± 6.5 years in nonsurvivors. The mean duration of CPR was 15.5 ± 6.6 min in survivors and 30.16 ± 6.6 min in nonsurvivors (P = 0.0001). Patient characteristics, ROSC, duration of CPR, FOUR scores before CA, and 1-week mean FOUR scores after ROSC are shown in Table 2. 

**Table 2:** Patient and resuscitation characteristics.

Patient no.	Sex	Age (years)	Etiology of CA	Prearrest chronic diseases	ROSC	Duration of CPR (min)	FOUR scores before CA	1-Week FOUR scores after ROSC (mean ± SD)
1	M	66	POIAI and sepsis	Ileus, hypertension	+	31	11	
2	M	71	Metabolic acidosis	CRF, hypertension	-	14	11	7.57 ± 0.5
3	F	80	Metabolic acidosis	Endometrial carcinoma	-	21	11	
4	M	78	Cardiac reasons	CHF, hypertension	+	8	12	7.57 ± 0.5
5	F	72	Trauma	-	+	15	12	8.14 ± 0.4
6	F	70	Unknown	Hypertension	+	21	10	6.50 ± 0.7
7	F	71	Metabolic acidosis	ARF, hypertension, DM	-	35	10	
8	M	81	Cardiac reasons	CHF, hypertension	-	38	12	
9	M	85	Sepsis	Parkinson disease	-	21	11	
10	F	68	Unknown	Hypertension	+	7	12	8.0 ± 0.6
11	F	75	Sepsis	CRF, hypertension	-	29	11	
12	F	78	Metabolic acidosis	Cirrhosis	-	25	11	
13	F	83	Metabolic acidosis	CRF, hypertension	-	33	10	
14	M	76	Cardiac reasons	CHF, hypertension	+	14	12	8.28 ± 0.5
15	M	77	Sepsis	DM	-	27	10	
16	F	68	POIAI, sepsis	DM, hypertension	+	18	11	8.0 ± 0.8
17	F	88	Sepsis	CRF, hypertension	-	25	11	
18	F	70	Unknown	Hypertension	-	36	12	
19	M	74	Cardiac reasons	CHF, hypertension	-	41	12	
20	F	78	CO poisoning	DM	+	27	12	8.85 ± 0.4

CA: Cardiac arrest, ROSC: return of spontaneous circulation, CPR: cardiopulmonary resuscitation, FOUR: Full Outline of Unresponsiveness, SD: standard deviation, POIAI: postoperative intraabdominal infection, CRF: chronic renal failure, CHF: congestive heart failure, ARF: acute renal failure, DM: diabetes mellitus, CO: carbon monoxide, M: male, F: female.

In ROSC and non-ROSC cases, the mean baseline (before CA) rSO2 values were 63.6 ± 3.3% and 62.8 ± 4.1%, minimum rSO2 values during resuscitation were 32.1 ± 4.6% and 26.9 ± 4.4%, maximum rSO2 values were 54.5 ± 2.4% and 45.0 ± 34.7%, and mean rSO2 values were 43.0±4.0% and 38.4±3.9%, respectively. Baseline, minimum, maximum, and mean rSO2 values for all patients are shown in Table 3. 

**Table 3:** Baseline, minimum, maximum, and mean rSO2 values for all patients.

Patient no.	ROSC	Baseline rSO2(before CA)	Min rSO2during CPR	Max rSO2during CPR	rSO2 during CPR(mean ± SD)	Patient no.	ROSC	Baseline rSO2(before CA)	Min rSO2during CPR	Max rSO2during CPR	rSO2 during CPR(mean ± SD)
1	-	55	24	43	32.3 ± 4.8	11	-	63	26	46	40.3 ± 5.0
2	+	68	30	53	37.8 ± 5.7	12	-	62	27	46	40.1 ± 5.0
3	-	69	30	40	34.6 ± 3.3	13	-	67	35	46	41.3 ± 2.6
4	+	65	35	58	41.3 ± 7.6	14	+	66	38	56	48.7 ± 5.3
5	+	60	33	52	43.2 ± 4.5	15	-	62	28	48	41.8 ± 4.9
6	+	58	24	48	39.7 ± 6.9	16	+	62	29	57	42.5 ± 7.8
7	-	60	32	51	41,9 ± 4.7	17	-	63	23	45	38,1 ± 6.6
8	-	58	22	46	38,1 ± 6.2	18	-	65	26	47	40,0 ± 6.0
9	-	68	20	37	30,0 ± 4.1	19	-	61	30	50	41,4 ± 5.1
10	+	65	31	52	41,8 ± 7.9	20	+	65	37	56	48,9 ± 4.7

CA: Cardiac arrest, rSO2: regional cerebral oxygen saturation, ROSC: return of spontaneous circulation, CPR: cardiopulmonary resuscitation, SD: standard deviation. Min: minimum, Max: maximum.

**Figure 1:**
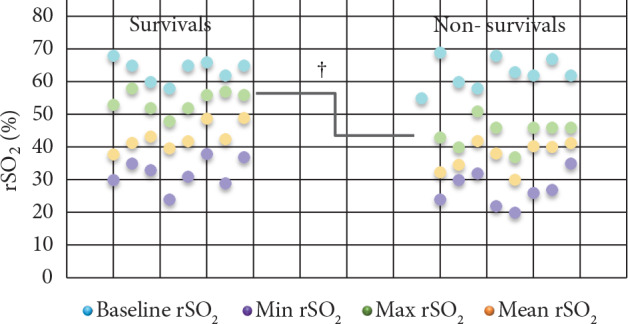
The scatterplot of basal, minimum, maximum, and mean rSO2 values for survivors and nonsurvivors. ϯ: Maximum rSO2 values were higher in survivors than in nonsurvivors (P = 0.005). rSO2: Regional cerebral oxygen saturation. Min: Minimum, Max: maximum.

There was no significant difference between survivors and nonsurvivors in terms of basal, minimum, and mean rSO2 values, whereas maximum rSO2 values were higher in survivors than in nonsurvivors (P = 0.005). The scatterplots of basal, minimum, maximum, and mean rSO2 values of survivors and nonsurvivors are shown in Figure 1. 

The mean FOUR score before CA was 11.50 ± 0.8 in survivors, whereas this value was 7.87 ± 0.7 for 1 week after ROSC (P < 0.0001).

**Figure 2:**
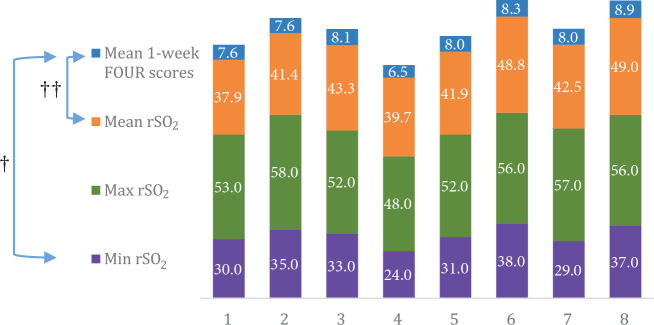
The minimum, maximum, and mean rSO2 (%) values and mean 1-week FOUR scores of each survivor. † Positive correlation between the minimum rSO2 values during CPR and the mean 1-week FOUR scores (r = 0.811, P = 0.015). †† Positive correlation between the mean rSO2 values during CPR and the mean 1-week FOUR scores (r = 0.771, P = 0.025). FOUR: Full Outline of Unresponsiveness, Min: minimum, Max: maximum.

There was a significant positive correlation between the minimum and mean rSO2 values during CPR and the mean 1-week FOUR scores in survivors (r = 0.811, r = 0.771 and P = 0.015, P = 0.025, respectively). The minimum, maximum, and mean rSO2 values and mean 1-week FOUR scores of each survivor are shown in Figure 2.

## 4. Discussion

Although there are many studies showing that NIRS reflects cerebral oxygenation well, we have not encountered a study that investigates the neurological outcomes with quality CC in humans. Findings in this study showed that the rSO2 values measured by NIRS are closely related to the state of consciousness and survival of patients with ROSC.

The interval between collapse and start of CPR is one of the first and most important factors affecting the neurological status of survivors (13). It is challenging to determine this interval, especially in CA cases coming from outside of a hospital into emergency services. As the CPR duration is prolonged, optimal cerebral perfusion cannot be achieved until the underlying cardiac defect improves and consequently prolonged CPR is associated with poor neurological outcome (14,15). Kim et al. carried out a study about the optimal transition time to extracorporeal CPR for predicting good neurological outcome in patients with out-of-hospital CA and they stated that good neurological outcomes were significantly reduced 21 min after CA (16). All our patients in our study had a fast onset of CPR due to follow-up in the ICU.

The mean duration of CPR in our study was 15.5 ± 6.6 min in survivors. There was no significant correlation between CPR duration and 1-week FOUR scores (P = 0.699). Unlike in the literature, we observed that the good neurological status in survivors is mostly associated with minimum or mean rSO2 values during CPR rather than CPR duration. Recently, several studies have been conducted to confirm that rSO2 values are closely related to ROSC. Shewe et al. reported that higher rSO2 values during CPR were associated with ROSC (17). Parnia et al. carried out a feasibility study evaluating the role of NIRS in patients with CA in the hospital and stated that the mean rSO2 values were significantly higher in survivors (35 ± 5%) than in nonsurvivors (18 ± 0.4%) (P < 0.001) (18). In our study, maximum rSO2 values were higher in survivors (54.5 ± 2.4%) than in nonsurvivors (45.0 ± 34.7%) (P = 0.005). This may be evidence that high rSO2 values during CPR may provide ROSC.

Many scoring methods can be used to assess neurological status. The cerebral performance category (CPC) is a more effective scoring method to assess long-term neurological outcomes and survival after CA (19). Sanders et al. used the CPC score for estimating good neurological outcomes in an experimental swine model (20). They investigated the best compression-ventilation rates for a good neurological outcome. According to their study, the 100:2-CPR model had the best neurological outcome. In our study, we investigated the effect of rSO2 values rather than compression-ventilation rates on neurological outcomes. Recently, the FOUR score has been a more commonly used scoring system to measure the depth of a coma. In our study, the mean FOUR score before CA was 11.50 ± 0.8 in survivors, whereas this value was 7.87 ± 0.7 after ROSC for 1 week (P < 0.0001). This was a natural result, since keeping the rSO2 values above the threshold of 50% to prevent cerebral ischemia throughout CPR is quite difficult even with good CC. In the study of Kämäräinen et al., rSO2 values were 28% (16%–33%) during high-quality CPR in hospital CA and they stated that frontal cerebral rSO2 remained low until ROSC (4).

**Figure 3:**
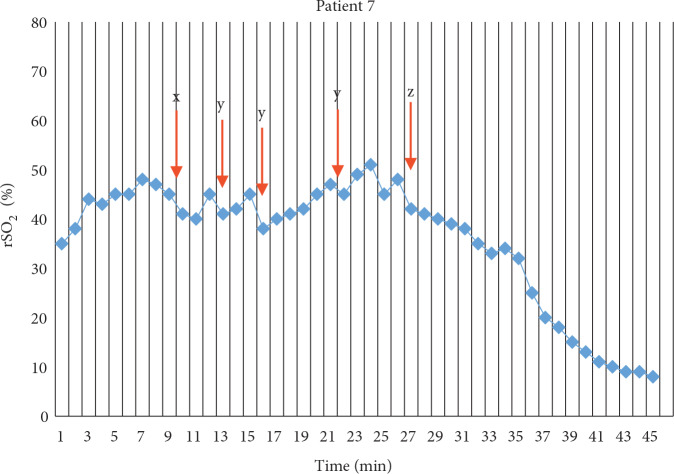
The rSO2 of patient 7 during CPR and after CPR. The caregiver who performed chest compressions for the 7th patient started the chest compressions at the 7th minute and another person took over the compression at the 12th minute. The decrease in rSO2 over this time period is indicated by x arrow. The y arrows indicate rSO2 decreases during frequent rhythm analysis. CPR was terminated at the point indicated by the z arrow.

Quality CPR is closely related to CC being sufficiently deep and uninterrupted. If CC is frequently interrupted for checking the pulse, this will reduce minimum and mean rSO2 values, resulting in neurological impairment. In our study, for the 7th patient, a person started CC at the 7th minute and another person took over the compression at the 12th minute. The decrease in rSO2 over this period is indicated by the x arrow in Figure 3. In the same graph, y arrows indicate rSO2 decreases during frequent rhythm analysis. Accordingly, Meex et al. showed that in their study, in which NIRS device technology was investigated, the rSO2 values were increased by switching the person giving CPR (21). Whatever the reason, impairment of the quality of CC causes simultaneous decrease of rSO2 values. In our study, survivors had a very strong positive correlation (r = 0.811, r = 0.771 and P = 0.015, P = 0.025, respectively) between the minimum rSO2 and mean rSO2 values and the 1-week mean FOUR scores. This result supports the close relationship between impairment of CC quality during CPR and poor neurological outcomes. 

This preliminary study had some limitations. First, we had a small number of patients. It is complicated to apply such technical monitoring in ICUs. Therefore, this study does not have extensive conclusions. However, we suggest that these results can be a guide as a pilot trial for further investigations about neurological outcomes after CPR. Second, the additional illnesses of patients might have affected post-resuscitation FOUR scores. Although we registered additional diseases of our patients, we could not measure the effectiveness of the diseases on FOUR. Finally, we used FOUR scoring for survival. There are many scoring systems to evaluate neurological status, but there is no scoring system for short-term prognosis aside from the Glasgow Coma Scale (GCS). However, many shortcomings of the GCS have been recognized. First, many comatose patients in ICUs are intubated and the verbal component cannot be evaluated. Second, the GCS does not include many indicators such as changing breathing patterns and abnormal brainstem reflexes. Third, the GCS may not detect vague changes during neurological examination. Therefore, we had to use only the FOUR scoring system for 1-week. 

In conclusion, although the results of this study were obtained from a limited number of patients, our results suggest that the maximum rSO2 values affect ROSC while the minimum and mean rSO2 values affect the post-cardiac arrest neurological outcome. Further studies including more subjects will strengthen the results of such studies.
